# Molecular basis of the phosphorothioation-sensing antiphage defense system IscS–DndBCDE–DndI

**DOI:** 10.1093/nar/gkae1133

**Published:** 2024-11-29

**Authors:** Yaqian Tang, Dan Wu, Yueying Zhang, Xuan Liu, Hui Chu, Qian Tan, Lixu Jiang, Shi Chen, Geng Wu, Lianrong Wang

**Affiliations:** Department of Gastroenterology, Ministry of Education Key Laboratory of Combinatorial Biosynthesis and Drug Discovery, Hubei Clinical Center and Key Laboratory of Intestinal and Colorectal Disease, TaiKang Center for Life and Medical Sciences, Zhongnan Hospital of Wuhan University, School of Pharmaceutical Sciences, Wuhan University, 169 Donghu Road, Wuchang District, Wuhan 430071, China; Department of Respiratory Diseases, Institute of Pediatrics, Shenzhen Children’s Hospital, Yitian Road 7019, Futian District, Shenzhen 518026, China; Senior Department of Nephrology, the First Medical Center of Chinese PLA General Hospital, Chinese PLA Institute of Nephrology, National Key Laboratory of Kidney Diseases, National Clinical Research Center for Kidney Diseases, Beijing Key Laboratory of Kidney Diseases Research, Fuxing road 28, Haidian District, Beijing 100853, China; Department of Gastroenterology, Ministry of Education Key Laboratory of Combinatorial Biosynthesis and Drug Discovery, Hubei Clinical Center and Key Laboratory of Intestinal and Colorectal Disease, TaiKang Center for Life and Medical Sciences, Zhongnan Hospital of Wuhan University, School of Pharmaceutical Sciences, Wuhan University, 169 Donghu Road, Wuchang District, Wuhan 430071, China; Department of Gastroenterology, Ministry of Education Key Laboratory of Combinatorial Biosynthesis and Drug Discovery, Hubei Clinical Center and Key Laboratory of Intestinal and Colorectal Disease, TaiKang Center for Life and Medical Sciences, Zhongnan Hospital of Wuhan University, School of Pharmaceutical Sciences, Wuhan University, 169 Donghu Road, Wuchang District, Wuhan 430071, China; Department of Gastroenterology, Ministry of Education Key Laboratory of Combinatorial Biosynthesis and Drug Discovery, Hubei Clinical Center and Key Laboratory of Intestinal and Colorectal Disease, TaiKang Center for Life and Medical Sciences, Zhongnan Hospital of Wuhan University, School of Pharmaceutical Sciences, Wuhan University, 169 Donghu Road, Wuchang District, Wuhan 430071, China; Department of Gastroenterology, Ministry of Education Key Laboratory of Combinatorial Biosynthesis and Drug Discovery, Hubei Clinical Center and Key Laboratory of Intestinal and Colorectal Disease, TaiKang Center for Life and Medical Sciences, Zhongnan Hospital of Wuhan University, School of Pharmaceutical Sciences, Wuhan University, 169 Donghu Road, Wuchang District, Wuhan 430071, China; Department of Burn and Plastic Surgery, Shenzhen Key Laboratory of Microbiology in Genomic Modification & Editing and Application, Shenzhen Institute of Translational Medicine, Medical Innovation Technology Transformation Center of Shenzhen Second People’s Hospital, Shenzhen University Medical School, The First Affiliated Hospital of Shenzhen University, Guanguang Road 1301, Longhua District, Shenzhen 518035, China; Department of Gastroenterology, Ministry of Education Key Laboratory of Combinatorial Biosynthesis and Drug Discovery, Hubei Clinical Center and Key Laboratory of Intestinal and Colorectal Disease, TaiKang Center for Life and Medical Sciences, Zhongnan Hospital of Wuhan University, School of Pharmaceutical Sciences, Wuhan University, 169 Donghu Road, Wuchang District, Wuhan 430071, China; Department of Burn and Plastic Surgery, Shenzhen Key Laboratory of Microbiology in Genomic Modification & Editing and Application, Shenzhen Institute of Translational Medicine, Medical Innovation Technology Transformation Center of Shenzhen Second People’s Hospital, Shenzhen University Medical School, The First Affiliated Hospital of Shenzhen University, Guanguang Road 1301, Longhua District, Shenzhen 518035, China; State Key Laboratory of Microbial Metabolism, School of Life Sciences and Biotechnology, Joint International Research Laboratory of Metabolic and Developmental Sciences, Shanghai Jiao Tong University, Dongchuan Road 800, Minhang District, Shanghai, 200240, China; Department of Gastroenterology, Ministry of Education Key Laboratory of Combinatorial Biosynthesis and Drug Discovery, Hubei Clinical Center and Key Laboratory of Intestinal and Colorectal Disease, TaiKang Center for Life and Medical Sciences, Zhongnan Hospital of Wuhan University, School of Pharmaceutical Sciences, Wuhan University, 169 Donghu Road, Wuchang District, Wuhan 430071, China; Department of Respiratory Diseases, Institute of Pediatrics, Shenzhen Children’s Hospital, Yitian Road 7019, Futian District, Shenzhen 518026, China

## Abstract

Phosphorothioation serves as a DNA backbone modification mechanism, wherein a sulfur atom substitutes the nonbridging oxygen atom within the phosphodiester, facilitated by the gene products of *dndABCDE* or *sspABCD*. The combination of *dndABCDE* with *dndFGH* forms a bona fide defense system, where the DndFGH protein complex exhibits DNA nickase and DNA translocase activities to prevent phage invasion. In this study, we identified that *dndI*, co-transcribed with *dndFGH*, can independently couple with *iscS-dndBCDE* as an anti-phage defense system. Moreover, we resolved the crystal structure of DndI from *Salmonella* at a resolution of 3.10 Å. We discovered that its residue Y25, residing within a hydrophobic region of DndI, is involved in phosphorothioate (PT) sensing. Upon sensing PT modifications at 5′-G_PS_AAC-3′/5′-G_PS_TTC-3′, the ATPase activity of DndI is stimulated, which subsequently triggers a conformational transition, facilitating the dissociation of DndI from self-DNA, thereby allowing DndI to avoid cleaving self-DNA while restricting PT-deficient phage DNA. This research broadens the knowledge of the mechanistic diversity underlying PT-based defense systems and highlights their complexity in the course of evolution.

## Graphical Abstract

**Graphical Abstract:**
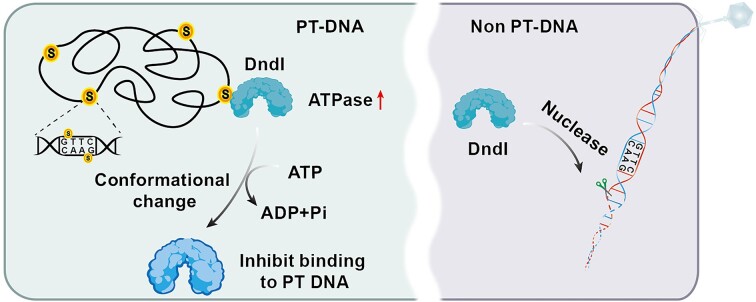

## Introduction

Bacteria and their viral invaders, known as bacteriophages or simply phages, are locked in a continuous arms race. Each side continuously evolves sophisticated countermeasures against the other. With an estimated 10^31^ phages on our planet, they far outnumber their bacterial hosts by a factor of 10 (1). Faced with such overwhelming numbers, bacteria have developed an array of defense strategies to fend off phage infection. These include, but are not limited to CRISPR-Cas systems (2,3), restriction-modification (R-M) systems (4,5), abortive infection (6–10), bacteriophage exclusion (11,12) and DNA phosphorothioate (PT) modification-based defense systems (13–17).

PT modification-based defense systems are composed of two contrasting enzymatic activities that functionally mirror the methyltransferase and restriction endonuclease in DNA methylation-based R-M systems (13,18–20). In PT-based Dnd defense systems, DndABCDE serves as the modification component to catalyze double-stranded DNA PT modification at the 5′-G_PS_AAC-3′/5′-G_PS_TTC-3′ sites (PS, phosphate-sulfur linkage), while DndFGH utilizes sequence-specific PT modification as the recognition tag to discriminate against and eliminate invasive foreign DNA lacking PT modification (21). It should be noted that in some strains, the function of DndA as a cysteine desulfurase can be functionally substituted by an IscS orthologue (22,23). Therefore, the PT modification module in Dnd defense systems can take the form of either *dndABCDE* or *iscS-dndBCDE*. Upon phage invasion, DndF, DndG and DndH form a complex and exhibit DNA nickase and DNA translocase activities to defend against invading genetic elements (21). Furthermore, in the halophilic archaeon *Haloterrigena jeotgali* A29, DndCDEA-catalyzed DNA PT modifications couple with the PbeABCD module to offer protection against archaeal viruses (24). In contrast to DndABCDE or IscS-DndBCDE in Dnd systems, the modification constituent SspABCD in PT-based Ssp defense systems provides single-stranded DNA PT modification at the 5′-C_PS_CA-3′/5′-TGG-3′ motifs, and combines with SspE or SspFGH to form the SspABCD-SspE or SspABCD-SspFGH antiphage systems (25,26). Given that the sulfur atom in the PT linkage is negatively charged and more hydrophobic than the equivalent oxygen present in native phosphodiester, SspE has evolved a hydrophobic cavity capable of interacting with 5′-C_PS_CA-3′/5′-TGG-3′ containing DNA, thereby modulating its GTPase and nickase activities. The antiphage activity of SspE relies on the presence of 5′-C_PS_CA-3′/5′-TGG-3′ modification in self-DNA, making SspABCD-SspE a PT-sensing defense barrier against phages (27).

Although it has been confirmed that *iscS-dndBCDE* and *dndFGH* form an antiphage defense system, in this study, we found that a *dndI* gene, situated within the *dndFGHI* operon, also constitutes a PT-based antiphage defense system along with the *iscS-dndBCDE* module. We formerly reported that SspE serves as a PT reader via a hydrophobic patch, subsequently triggering its GTPase activity, facilitating the closed-to-open conformational shift and ultimately activating the C-terminal nickase activity to fend off phages (27). In the absence of 5′-C_PS_CA-3′/5′-TGG-3′ modification, SspE alone exhibits no toxic nuclease activity towards host cells (27). Based on structural and biochemical analyses, a tyrosine-containing hydrophobic patch was also identified in the N-terminal region of DndI, which governs the response to PT modification at 5′-G_PS_AAC-3′/5′-G_PS_TTC-3′. Contrary to SspE, which requires PTs to activate its nuclease activity, DndI exerts nuclease activity independent of PT modification and induces toxicity to PT-deficient cells. Furthermore, our data, in conjunction with the observations that the ATPase activity of DndI can be enhanced by PTs and ATP hydrolysis suppresses the DNA-binding activity of DndI, support a model in which PTs facilitate the dissociation of DndI from self-DNA, thus accounting for the prevention of DndI autoimmunity.

## Materials and methods

### Strains, plasmids and phages

The strains, plasmids and phages utilized in this study were described in detail in Supplementary Table S1. *Salmonella enterica* and its variants were cultivated in Luria–Bertani (LB) medium at 28°C. *Escherichia coli* cells were cultivated in LB medium at 37°C.

### Phage infection growth curves

After incubation overnight at 28°C, *S. enterica* serovar Cerro 87 was diluted 1:100 in 5 mL of LB once the OD_600_ of the cultures reached 0.6. Bacteriophages were added at various MOIs, and 200 μL of the mixture was added to each well of a 96-well plate. The growth was tracked with a Multiskan GO microplate spectrophotometer (Thermo Fisher Scientific), maintained at 28°C and stirred continuously. The OD_600_ was measured every 30 min for 24 h. LB medium served as a reference control.

### Plaque assays

To produce a bacterial lawn, 1 mL of JM109 culture with an OD_600_ of 0.6, was combined with 10 mL of 0.75% liquid top agar and then was uniformly distributed over a 1.5% LB agar plate. The phage suspensions were subjected to ten-fold serial dilution using SM buffer (100 mM NaCl, 50 mM Tris-HCl, pH 7.5 and 8 mM MgSO_4_). Subsequently, 3 μL from each dilution was added on the bacterial lawn. Plaque images were taken through ChemiDoc XRS+ (Bio-Rad) following a 12 h incubation period at 37°C.

### Protein expression and purification

The *S. enterica dndI* gene was amplified and inserted into the pET28a vector. The generated plasmid was subsequently introduced into *E. coli* BL21 (DE3) cells at 37°C. The OD_600_ of the cells reached 0.6 to 0.8. The cells were subsequently cooled to 16°C and induced with 0.4 mM isopropyl β-D-1-thiogalactopyranoside. To promote protein expression, the cells were incubated overnight at 16°C. After overnight incubation, the cells were removed and resuspended in wash buffer, which contained 150 mM NaCl, 20 mM imidazole and 25 mM Tris-HCl (pH 8.0). The cells were then disrupted. The cell lysates were then centrifuged at 16, 000 g for 1 h at 4°C to remove the cell debris. The affinity chromatography of supernatant was mixed with Ni^2+^-NTA affinity beads (Yeasen), followed by elution with a buffer solution (25 mM Tris-HCl (pH 8.0), 150 mM NaCl and 200 mM imidazole). The eluted DndI protein was further purified by size exclusion chromatography using a HiLoad 16/600 Superdex 200 pg gel filtration column (GE Healthcare).

### ATPase assays

ATPase assays were carried out to evaluate the ATP hydrolysis activity of DndI and its variants using the Malachite Green Phosphate Detection Kit from Beyotime Biotechnology. 250 ng pUC19 DNA containing either the 5′-G_PS_AAC-3′/5′-G_PS_TTC-3′ or 5′-GAAC-3′/5′-GTTC-3′ motif was added in the reaction mixture as indicated. The reaction mixture was prepared in r3.1 buffer (100 mM NaCl, 50 mM Tris-HCl (pH 7.9), 10 mM MgCl_2_ and 100 μg/mL BSA) and incubated at 37°C for 1 h. Subsequently, 160 μL double-distilled water and 70 μL of the reagent included in the kit were added to the reaction solution. The mixture was left to stand at room temperature for 30 min to allow the development of the green phosphomolybdic acid complex. The amount of released inorganic phosphate was quantified by measuring the absorbance at 630 nm.

### DNA nicking assays

In the DNA nicking assays, either 250 ng of pUC19 DNA or 150 ng genomic DNA was incubated with 2 μM of DndI or variants in r3.1 buffer (New England Biolabs) in a 10 μL reaction mixture at 37°C. The reaction was quenched by the addition of 6 × DNA loading buffer (Yeasen) and analyzed using a 1% native agarose gel.

### Electrophoretic mobility shift assay

In a 10 μL reaction mixture, 100 nM of 70 bp DNA fragments were incubated with varying concentrations of DndI and its mutants in r3.1 buffer (New England Biolabs) at 4°C for 1 h. Subsequently, the samples were analyzed on 6% polyacrylamide gels. The data were quantified via Image Lab software, and curve fitting was conducted via GraphPad Prism 8.0.

### Crystallization and structure determination

Crystals of DndI from *S. enterica* serovar Cerro 87 were grown using the hanging-drop vapor-diffusion method in a buffer containing 4% PEG6000 and 100 mM MES (pH 6.1) and maintained at 4°C. Crystals were cryoprotected using a solution containing 25% glycerol. Crystal diffraction data at 100 K were collected at the BL19U1 beamline in National Center for Protein Sciences (Shanghai). The data were then analyzed using HKL3000 software and the resolution was found to be 3.10 Å. The DndI crystal structure was determined via the single-wavelength anomalous dispersion (SAD) approach and the PHASER program. COOT and REFMAC software were used for model building and refinement. The crystals, which have two DndI molecules in each asymmetric unit, belonged to the space group *C222_1_*. The crystal data was provided in Supplementary Table S2.

### FRET analysis

A 717 base pair segment encoding cyan fluorescent protein (CFP) was amplified using the primers CFP-F and CFP-R, and cloned into the pET28a plasmid using BamHI and NotI restriction enzymes. Similarly, the DNA encoding yellow fluorescent protein (YFP) was amplified using primers YFP-F and YFP-R. A 1, 788 bp fragment of the *dndI* gene was amplified from *S. enterica* serovar Cerro 87 using the primers DndI-YFP-F and DndI-YFP-R. The PCR products containing the YFP and DndI genes were fused through overlap extension PCR with primers YFP-F and DndI-YFP-R. After digestion with NdeI and BamHI, the resultant YFP-DndI fragment was ligated into pET28a-CFP, generating the construct pPT010. The FRET assays were performed using the YFP-DndI-CFP proteins at final concentration of 0.2 μM in r3.1 buffer with an Infinite 200 PRO (TECAN). The FRET emission fluorescence spectra were detected at wavelength ranging from 450 to 600 nm after excitation at 430 nm. The sequences of primers utilized in this research are provided in Supplementary Table S3.

### Bioinformatics analysis

A total of 31, 478 fully sequenced bacterial and archaeal genomes were retrieved from the NCBI RefSeq database (November 2023). Utilizing the phosphoadenosine phosphosulfate reductase gene (*dndC*) as an anchor, this gene along with 15 upstream and 15 downstream Open Reading Frames (ORFs) was extracted for downstream analysis. Subsequently, the DndBCDFGHI seven-protein sequences were employed as queries to perform BLASTp searches against the NCBI non-redundant protein database. DndE was not included in this search protocol because this protein is so small that it cannot be annotated in many genomes. From the resulting hits, the top 100 protein sequences were selected to construct hidden Markov model (HMM) profiles for each protein using HMMER3 software v3.4 (28). These HMM profiles were then utilized in hmmsearch to scan the extracted protein sequences, with an e-value threshold of 0.0001. The data were provided in Supplementary Table S4.

## Results

### IscS-DndBCDE and DndI constitute a new type of PT-based antiphage system

**Figure 1.**
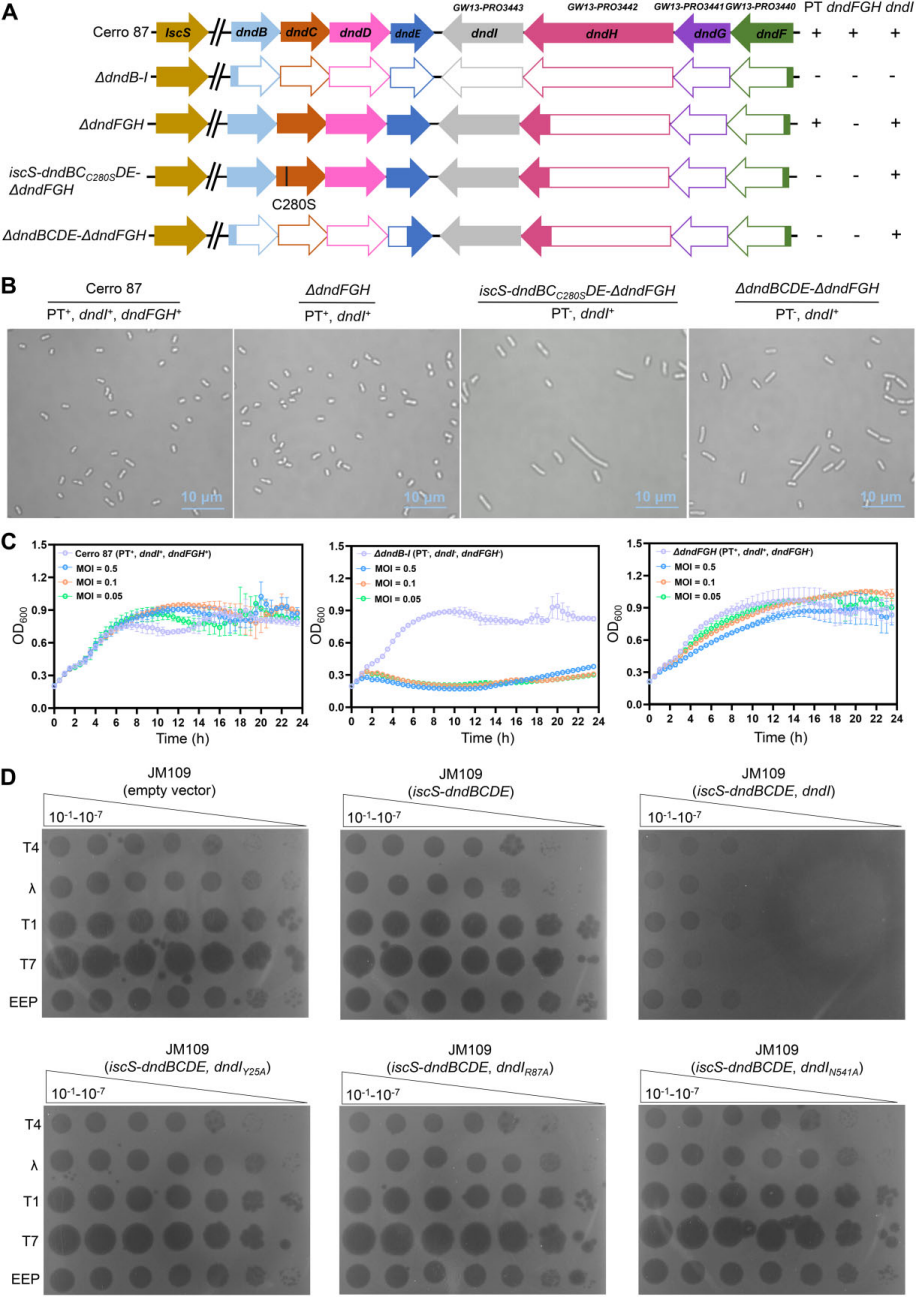
The IscS-DndBCDE and DndI form a defense system. (**A**) Schematic representation of the deletion mutants generated. The white boxes indicate in-frame deletions in the respective genes. (**B**) Cell morphology of wild-type *S. enterica* serovar Cerro 87 and mutants expressing combinations of *dnd* genes. Cell elongation was noticed in the *dndI*-expressing, PT-lacking *S. enterica* mutants, including *iscS-dndBC*_*C280S*_*DE-ΔdndFGH* and *ΔdndBCDE*-*ΔdndFGH*. (**C**) Growth curves of Cerro 87 and its mutants treated with phage PT1 at the indicated MOIs. (**D**) Phage plaque assays on *E. coli* JM109 strains carrying empty vectors, *iscS-dndBCDE* or *iscS-dndBCDE-dndI* or variants from Cerro 87 using 3 μL each of ten-fold serial dilutions (10^−1^-10^−7^) of phages. No phage resistance was detected when only DndBCDE was expressed in *E. coli* JM109.

Previous studies have shown that in *S. enterica* serovar Cerro 87, IscS and DndBCDE work together to provide DNA with PT modification at the 5′-GAAC-3′/5′-GTTC-3′ sites, yielding 5′-G_PS_AAC-3′/5′-G_PS_TTC-3′ and couple with DndFGH to confer phage resistance in a manner similar to methylation-based R-M systems (21,29). In the absence of PT modification, DndFGH is unable to differentiate between self-DNA and non-self-DNA, consequently causing harm to the cells (30,31). The PT modification-deficient *S. enterica* mutant shows a growth defect and a cell filamentation phenotype because of the DNA damage caused by the unrestrained nuclease activity of DndFGH (30,31). Notably, a 1, 971 bp ORF, namely *dndI*, has been found adjacent to the *dndBCDE* operon and cotranscribed with the *dndFGH* genes, yet its function remains enigmatic (31) (Figure 1A). Sequence analysis reveals that DndI comprises an N-terminal DUF262 and a C-terminal DUF1524 His-Asn-His (HNH) nuclease domain, similar to the domain organization in SspE (25). The DUF262 domain belongs to the ParB-like superfamily, which encompasses diverse proteins such as ParB, Osa, SspE, DndB and sulfiredoxin, implicated in a broad spectrum of biological contexts (32–35). The HNH nuclease domain belongs to the His-Me finger endonuclease superfamily and harbors a conserved HNH motif, folding in a ββα-metal topology (36,37). Intriguingly, the cell filamentation phenotype persisted even when both *dndBCDE* and *dndFGH* genes were deleted (Figure 1A and B). In contrast, no such phenotype was observed in the *ΔdndB-I* mutant with all *dndBCDE-dndI-dndFGH* deleted, suggesting a correlation between DndI and cell elongation (Figure 1A and B). To delve deeper into this finding, a cysteine-to-serine single-point mutation was introduced at C280 in DndC. C280 is indispensable for coordinating the [4Fe-4S] cluster in DndC (38). Indeed, liquid chromatography-coupled tandem quadrupole mass spectrometry (LC-MS/MS) demonstrated that the *iscS-dndBC_C280S_DE*-*ΔdndFGH* mutant lost DNA PT modification (Supplementary Figure S1). Despite the maintenance of the integrity of the PT modification proteins in *iscS-dndBC_C280S_DE*-*ΔdndFGH*, the cells still exhibited growth delay and a filamentous phenotype (Figure 1B and Supplementary Figure S2). The data supported that DndI are detrimental to cells without DNA PT modification.

Based on these observations, we postulated that DndI, in association with IscS-DndBCDE, forms a potential PT-based defense system. To confirm this hypothesis, we subjected the wild-type Cerro 87 and mutants including *ΔdndFGH* and *ΔdndB-I* to the lytic phage PT1 (GenBank accession No. MT012302) at multiplicities of infection (MOI) of 0.05, 0.1 and 0.5. Growth curves revealed that at an MOI of 0.5, the *ΔdndFGH* mutant proliferated normally, while the *ΔdndB-I* mutant underwent lysis at an MOI of 0.05 (Figure 1A and C). To measure the protective effect, the phage efficiency of plating (EOP) of *iscS-dndBCDE-dndI*-expressing *ΔdndFGH* against phage PT1 was assessed. The PT-based DndI module provided protection, albeit to a certain limit, as evidenced by the observation that the EOP on *ΔdndFGH* relative to *ΔdndB-I* was 0.8 ± 0.04 (Supplementary Figure S3A and B, Supplementary Table S5). The defense function of IscS-DndBCDE-DndI was further verified by the discovery that the average burst size of phage PT1 decreased from 72.5 ± 3.24 on infection of *ΔdndB-I* cell to 50.35 ± 1.17 on infection of *ΔdndFGH* cell (Supplementary Figure S3C and D). Nevertheless, the weak resistance to phage PT1 presented an obstacle in our attempt to analyze the defensive mode of DndI. As an alternative, we cloned the *dndBCDE* operon and *dndI* gene, under the regulation of their native promoters, into the pBluescript II SK(+) plasmid, generating plasmid pPT1002. This plasmid was then transformed into *E. coli* JM109 that contained chromosomally encoded *iscS*. Subsequently, we challenged JM109 (pPT1002) cells with phages T1, T4, T7, EEP and λ; the cells exhibited varying degrees of resistance to these phages (Figure 1D). Furthermore, we noticed that the deletion of *dndI* (leaving only the *dndBCDE* locus on the pPT1001 plasmid) abolished the protection against phage infection (Figure 1D). These data implied that *dndI*, despite being part of the *dndFGHI* operon, also establishes a PT-based defense system together with *iscS-dndBCDE* to protect against phage attack.

Upon analyzing 31, 478 fully sequenced bacterial and archaeal genomes, it was determined that 568 genomes possessed *dndBCD*. Since DndE is too small to be annotated in many genomes, it was not included in this search protocol. Additionally, 340 of these *dndBCD*-containing genomes were found to have *dndFGH* genes in close proximity to *dndBCD*. Notably, 61 genomes were discovered to have the *dndBCD* genes along with *dndI*, but without *dndFGH*. Further investigation revealed that 72 strains concurrently carried *dndBCD*, *dndFGH*and *dndI* (Supplementary Table S4). Given this, we were prompted to explore the molecular mechanism underlying PT-based DndI defense.

### DndI harbors NTPase and nickase activities

**Figure 2.**
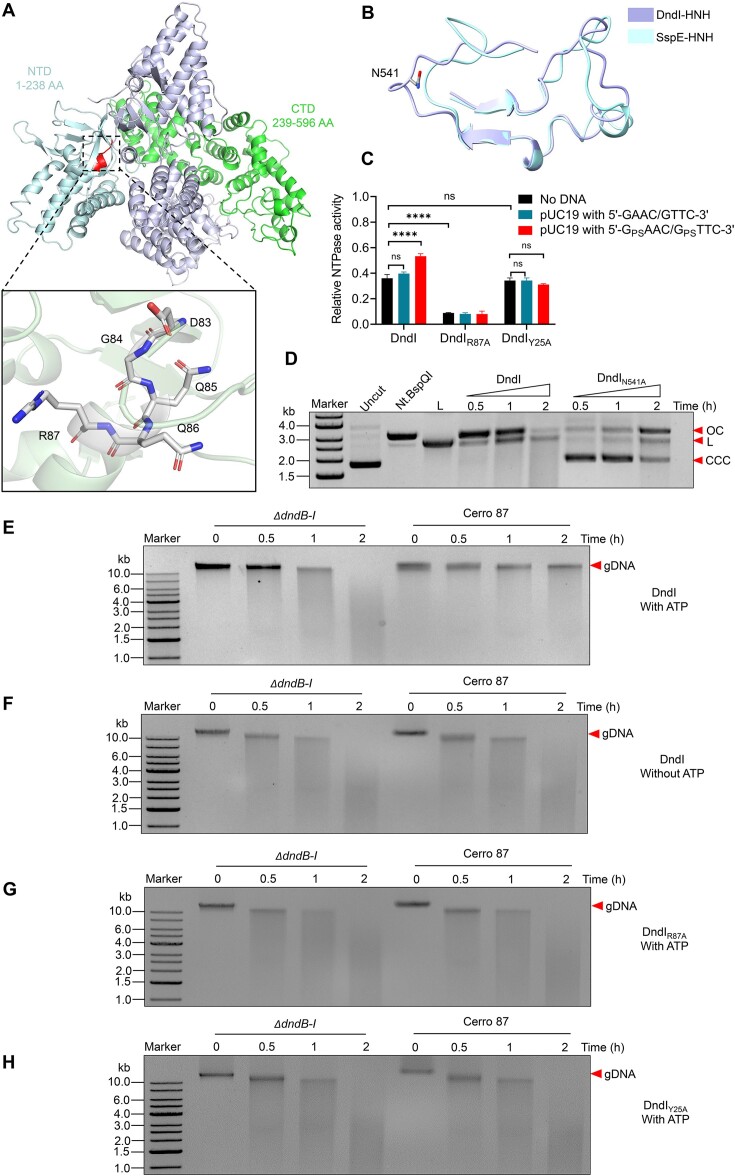
Structural and biochemical analysis of DndI. (**A**) A ribbon diagram depicts the full-length DndI dimer. In one promoter, the N-terminal DUF262 domain (comprising residues 1 to 238) and the C-terminal HNH domain (extending from residues 239 to 596) are respectively colored palecyan and green. The D^83^GQQR^87^ motif in the NTD is indicated in red, with a close-up view highlighting the residues. (**B**) Superposition of the HNH motifs from DndI (PDB: 9JFL, lightpurple) and SspE (PDB: 7DRS, lightcyan). (**C**) Evaluation of the ATPase activities of DndI and its mutants in the presence of 5′-G_PS_AAC/G_PS_TTC-3′ or 5′-GAAC/GTTC-3′ containing pUC19 DNA. Statistical significance was calculated by unpaired two-sided Student’s *t* tests. ****P < 0.0001. ns, not significant. (**D**) *In vitro* nicking nuclease activity assays of DndI and variants using supercoiled pUC19 as the substrate. The DNA products were analyzed on a 1% agarose gel. BamHI-linearized pUC19 and Nt. BspQI-nicked pUC19 served as references. CCC, covalently closed circular DNA; OC, open circular DNA; and L, linear DNA. All the results are representative of three independent experiments. (**E** and **F**) Time course of DndI-treated 5′-G_PS_AAC-3′/5′-G_PS_TTC-3′-containing Cerro 87 DNA and PT-lacking *ΔdndB-I* DNA in the presence or absence of 1 mM ATP. (**G**) Time course of DndI_R87A_-treated 5′-G_PS_AAC-3′/5′-G_PS_TTC-3′-containing Cerro 87 DNA and PT-lacking *ΔdndB-I* DNA in the presence of 1 mM ATP. (**H**) Time course of DndI_Y25A_-treated 5′-G_PS_AAC-3′/5′-G_PS_TTC-3′-containing Cerro 87 DNA and PT-lacking *ΔdndB-I* DNA in the presence of 1 mM ATP. The DNA products were analyzed on 1% agarose gels. All the results are representative of three independent experiments.

To elucidate the underlying defense mechanism of DndI function, we determined the crystal structure of full-length DndI from Cerro 87 to a resolution of 3.10 Å via SAD with the selenomethionine substituted protein (PDB accession number: 9JFL). In each asymmetric unit, DndI forms a homodimer, where each protomer of DndI consists of a smaller N-terminal domain (residues 1–238, hereinafter DndI_NTD_) and a larger C-terminal domain (residues 239–596, hereinafter DndI_CTD_) (Figure 2A and Supplementary Figure S4). The conserved D^83^GQQR^87^ motif, a key motif for NTP hydrolysis (27), is located at the junction of the α3 helix and the β3 sheet of DndI_NTD_ (Figure 2A). Unlike the typical HNH motif observed in Colicin E9 (39), ScoMcrA (40), Cas9 (41), BrxU (42) and Ref (43), which is composed of an α-helix and two antiparallel β-strands, the HNH motif located in DndI_CTD_ consists of two β-strands and a loop, showing similarities to the HNH motif found in SspE of *Streptomcyes yokosukanensis* DSM 40224 (27) (Figure 2B and Supplementary Figure S5A).

The results of the nucleoside triphosphatase assay indicated that DndI exhibited an intrinsic ATPase activity, which was enhanced by DNA containing 5′-G_PS_AAC-3′/5′-G_PS_TTC-3′ modification. Conversely, the ATP hydrolysis activity of DndI was not affected by PT-free pUC19 DNA (Figure 2C and Supplementary Figure S6). In addition, DndI demonstrated weak hydrolytic capabilities with regard to GTP, CTP and UTP (Supplementary Figure S6). The single substitution of R87 with alanine in the D^83^GQQR^87^ motif abolished the ATP hydrolysis activity of DndI_R87A_*in vitro* (Figure 2C). Meanwhile, the plaque assay results revealed that the R87A mutation led to a significant reduction in phage resistance compared to cells with wild-type IscS-DndBCDE-DndI, thereby confirming that the ATPase activity of DndI is crucial for phage defense (Figure 1D).

The existence of the HNH nuclease domain in DndI prompted us to study the nuclease activity of DndI. A time-course experiment showed that DndI behaved as a nickase, converting supercoiled pUC19 into an open circular form and eventually linearizing it (Figure 2D). At a concentration of 10 mM of divalent cations, DndI was more active with Mg^2+^ and Mn^2+^ than with other metal ions, such as Zn^2+^, Ni^2+^, Cu^2+^ and Ca^2+^ (Supplementary Figure S5B). As anticipated, the *in vitro* nuclease activity of DndI_N541A_, with a single substitution of N541 by alanine in the conserved HNH motif, was significantly decreased (Figure 2D). Consistent with these results, the plasmid-encoded DndBCDE-DndI_N541A_ no longer conferred protection to *E. coli* JM109 against phages, confirming the crucial role of the nuclease activity of DndI in phage defense (Figure 1D).

### PT modification regulates the ATPase activity of DndI

**Figure 3.**
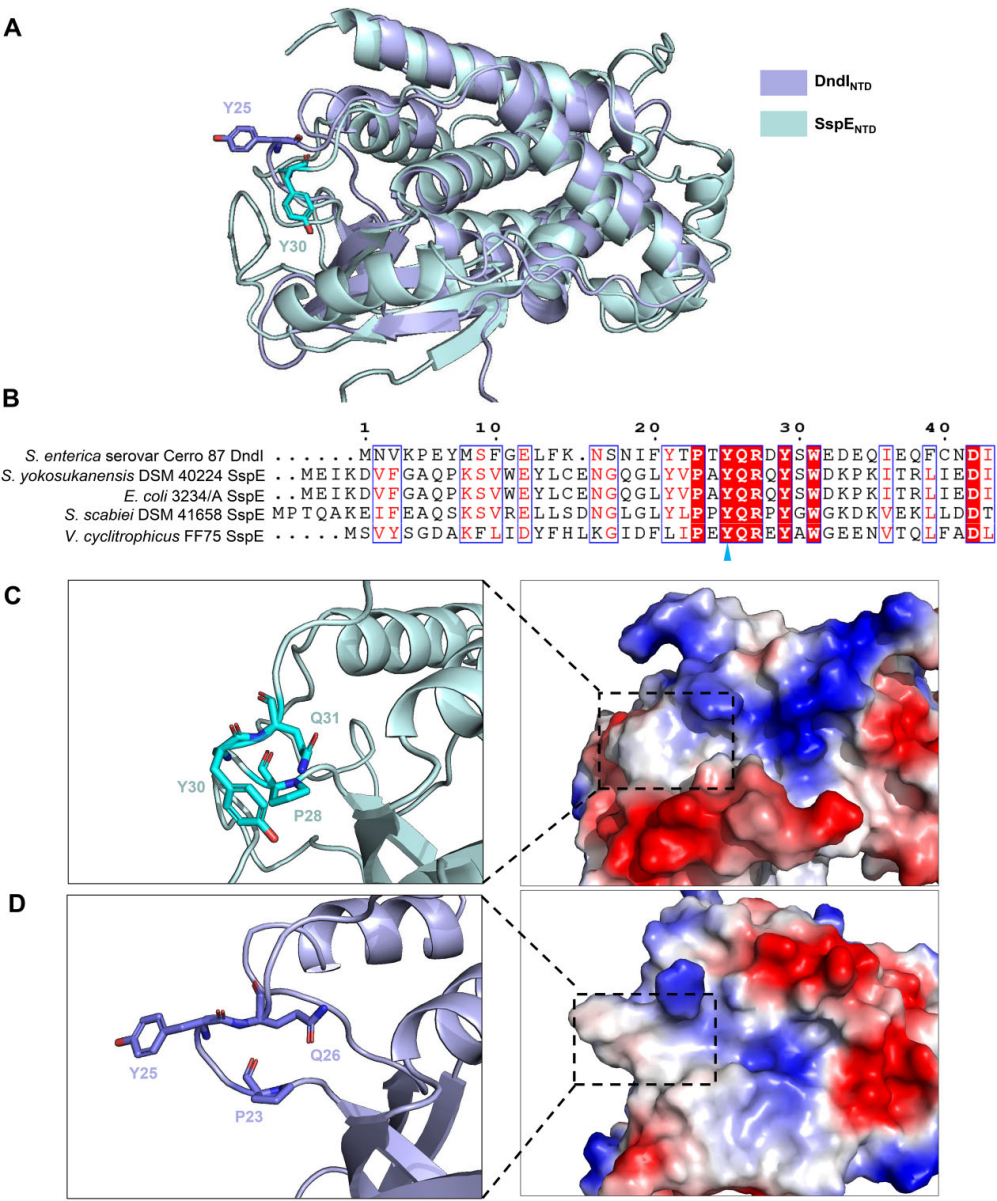
The PT-sensing hydrophobic patch in DndI. (**A**) Superimposition of DndI_NTD_ (lightblue, PDB code: 9JFL) and SspE_NTD_ (palecyan, PDB code: 7DRS), with Y25 and Y30 in DndI and SspE, respectively, represented by sticks in the structures. (**B**) Multiple sequence alignment of the Y25-containing regions of DndI from *S. enterica* serovar Cerro 87 (APT80315.1) with the corresponding ones of SspE from *S. yokosukanensis* DSM 40224 (WP_067135523.1), *Vibrio cyclitrophicus* FF75 (ERM61718.1), *E. coli* 3234/A (WP_000402995.1) and *Streptomyces scabiei* DSM 41658 (KFG02858.1). The conserved Y25 residue is indicated by blue arrow. (**C** and **D**) Close-up views of the PT-sensing hydrophobic patches from SspE and DndI, respectively. P23, Y25 and Q26 in DndI and their equivalents in SspE are displayed by sticks in the structures.

Notably, the *in vitro* nuclease assays demonstrated that DndI was unable to distinguish between 5′-G_PS_AAC-3′/5′-G_PS_TTC-3′-containing and 5′-G_PS_AAC-3′/5′-G_PS_TTC-3′-lacking DNA molecules, except when ATP was added to the reaction mixture (Figure 2E and F). Additionally, the ATPase-deficient DndI_R87A_ mutant is no longer capable of discriminating between PT-containing and PT-lacking DNA even in the presence of ATP (Figure 2G). In combination with the observation that the intrinsic ATPase activity of DndI was stimulated by PT DNA, we were inspired to investigate how the epigenetic PT markers modulate the defensive action of DndI. The CTDs of DndI and SspE exhibit a lower extent of structural similarity (with an RMSD of 4.9 Å for 325 Cα atoms) in comparison to the NTDs (with an RMSD of 3.0 Å for 219 Cα atoms) (Supplementary Figure S7). Taking this into account, along with the sensitivity of DndI to PT modification, we hypothesized the presence of a PT-sensing region in the NTD of DndI, similar to that in SspE (Figure 3A). Significantly, the Y25 residue, which is equivalent to Y30 that mediates the PT responsiveness in SspE from *S. yokosukanensis* DSM 40224, was situated in a hydrophobic cavity within the N-terminal ATPase domain of DndI (Figure 3B–D). The immediate finding was that the DndI_Y25A_ variant with a single-point alanine substitution at Y25 still exhibited intrinsic ATP hydrolysis activity comparable to wild-type DndI but was no longer enhanced by PT modification, suggesting that the Y25-containing hydrophobic patch mediates the responsiveness of DndI to PT markers (Figure 2C). This was further strengthened by two observations: DndI_Y25A_ was incapable of differentiating between PT-containing Cerro 87 DNA and PT-lacking *ΔdndB-I* DNA *in vitro*, even in the presence of ATP (Figure 2H), and the plasmid-encoded DndBCDE-DndI_Y25A_ module lost its resistance against phages (Figure 1D). These outcomes suggested that the Y25-containing hydrophobic cavity governs the response of DndI to PT modification, which in turn regulates the ATPase and nuclease activities of DndI to carry out the anti-phage defense.

### ATP hydrolysis modulates conformational change of DndI

**Figure 4.**
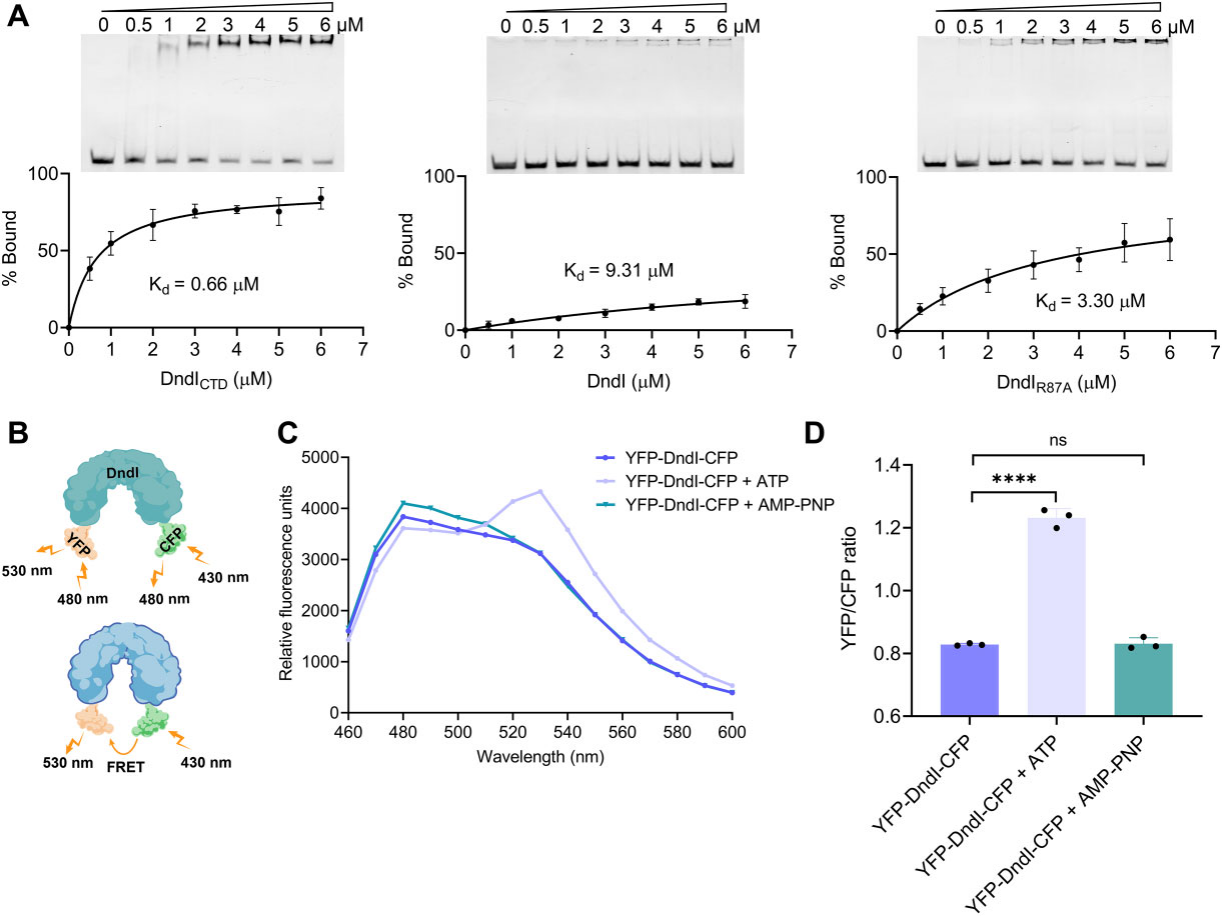
Effects of ATP on the conformational change and biochemical activities of DndI. (**A**) Evaluation of DNA binding via EMSA. The intensity of the shifted DNA band was presented as the percentage bound. Nonlinear regression analysis was implemented on the datasets through GraphPad Prism software (version 8.0) to acquire the optimal fit curve. The values represent the means of three replicates, and the error bars represent standard deviations. (**B**) A schematic illustration delineating the FRET alterations of YFP-DndI-CFP in the open to closed transition. In the closed state, FRET occurs from excited CFP to YFP, resulting in light emission from YFP at 530 nm. (**C**) Spectra of FRET monitoring of YFP-DndI-CFP *in vitro* in the presence of ATP and AMP-PNP. (**D**) FRET analysis of YFP-DndI-CFP *in vitro*. FRET was represented as the emission ratio of YFP to CFP signals. Data and error bars represent the mean ± SD from three independent experiments. Statistical significance was calculated by unpaired two-sided Student’s t tests; ***P< 0.001, ****P< 0.001 and ns, not significant.

To further understand how PT modification modulates other enzymatic activities of DndI, electrophoretic mobility shift assay (EMSA) was conducted, which showed that the binding affinity of DndI_CTD_ with DNA (*K*_d_ = 0.66 μM) was 14-fold greater than that of full-length DndI (*K*_d_ = 9.31 μM) (Figure 4A), indicating the inhibitory impact of the NTD on the CTD. In addition, the single-point mutation R87A, which abolished the ATPase activity of DndI, exhibited higher DNA binding affinity (*K*_d_ = 3.30 μM) than the full-length DndI (*K*_d_ = 9.31 μM) (Figure 4A). Based on these results, we postulated that the impact of NTD on the DNA-binding affinity of DndI is modulated by the ATPase activity of the NTD, maybe via a conformational change. To test this hypothesis, we attached a fluorescence acceptor YFP to the N-terminus and a fluorescence donor CFP to the C-terminus of the full-length DndI (Figure 4B). This resulting YFP-DndI-CFP still retained the ATP hydrolysis activity similar to that of DndI (Supplementary Figure S8A). Notably, in the presence of ATP hydrolysis, a fluorescence resonance energy transfer (FRET) signal, as shown by an increase in the YFP emission and a decrease in the CFP emission of YFP-DndI-CFP, was detected, suggesting a shorter distance between the NTD and CTD (Figure 4C and D). In contrast, the addition of unhydrolyzable AMP-PNP did not lead to a change in FRET (Figure 4C and D). However, the YFP-DndI-CFP variant lost its sensitivity to PT modification, as shown by the result that the addition of PT-modified DNA did not affect the ATP hydrolysis activity of YFP-DndI-CFP (Supplementary Figure S8A). Subsequently, the addition of ATP, along with PT-modified or PT-free pUC19 DNA, increased the FRET efficiencies to a similar degree, and this degree was comparable to that observed with the addition of ATP alone (Supplementary Figure S8B). Upon verification that YFP-Dndl-CFP preserved the oligomeric state, even in the presence of diverse DNAs and ATP, similar to unlabeled Dndl, the lack of its response to PT might potentially arise from the structural impact of the introduced YFP and CFP groups on the interaction between PT and the hydrophobic patch (Supplementary Figure S8C). Although the YFP-DndI-CFP variant failed to demonstrate that PT-enhanced ATPase activity led to a distinct FRET change compared to the combination of YFP-DndI-CFP and ATP, these results verified that ATP hydrolysis by DndI regulates the distance between the NTD and CTD, thereby modulating the DNA-binding affinity of DndI.

**Figure 5.**
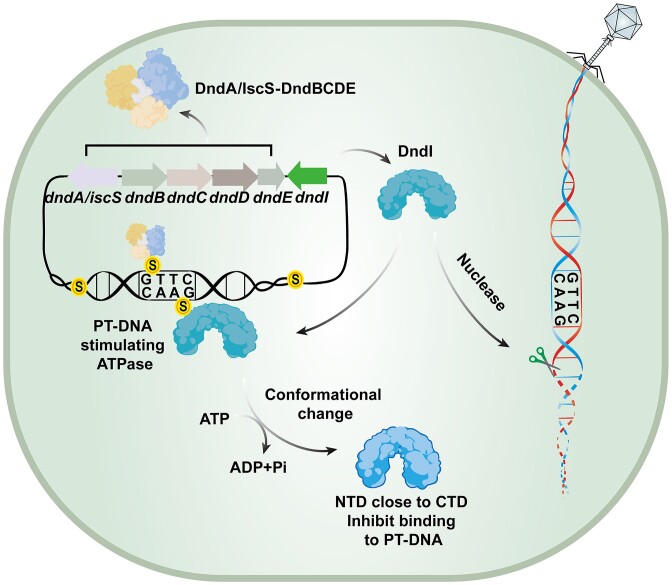
Schematic model of PT-based DndI defense against phage infection. The DndA/IscS and DndBCDE proteins act as the components responsible for introducing PT modifications at specific 5′-GAAC-3′/5′-GTTC-3′ sites within the host DNA, resulting in the formation of 5′-G_PS_AAC-3′/5′-G_PS_TTC-3′ sequences. DndI serves as the restriction component to induce nicking damages on PT-lacking phage DNA. Upon approaching PT sites, ATP hydrolysis of DndI is enhanced, followed by an open-to-closed transition and dissociation from the PT-modified bacterial genome, facilitating the avoidance of autoimmunity.

In summary, our findings imply a PT-responsive defense mechanism whereby DndI exerts nuclease activity within cells to eradicate PT-deficient invasive phage DNA. Upon DndI approaching self-DNA, PT modification enhances the ATPase activity of DndI, triggering a conformational shift from open to closed, presumably facilitating DndI’s dissociation from PT-modified self-DNA, thereby averting self-targeting (Figure 5).

## Discussion

In this research, IscS-DndBCDE-DndI was characterized as a new sort of PT-based defense system, presenting unique genetic architecture and phenotypic behavioral variances when compared with known PT-based defense modules such as DndBCDE-DndFGH, SspABCD-SspFGH and SspABCD-SspE (21,25,26). Both DndI and DndFGH differentiate between self-DNA and non-self-DNA by exploiting PT modifications at 5′-G_PS_AAC-3′/5′-G_PS_TTC-3′ as the recognition tag. Without PT protection, both DndI and DndFGH are detrimental to cells due to their unrestricted nuclease activities. DndF, DndG and DndH form a DndFGH complex in a 2:2:1 molecular ratio, and DndFGH exerts translocation-coupled nicking nuclease activity to harm phage DNA, thereby inhibiting phage proliferation. PT modifications at 5′-G_PS_AAC-3′/5′-G_PS_TTC-3′ repress the ATP hydrolysis activity of DndFGH, thereby impeding the translocation of DndFGH along PT-modified self-DNA, thus evading self-immunity (21). In contrast to DndFGH, the ATPase activity of DndI is enhanced by PTs. Nevertheless, the nuclease activity of both DndI and DndFGH remains regardless of the presence of PT modification, and PT modification serves a purpose in regulating the functionalities of DndFGH or DndI to prevent self-immunity. This could potentially explain the evolution of *dndI* and *dndFGH* into a single operon.

Particularly, a similar induction in NTPase activity initiated by PT modification has also been observed in the SspE protein. However, in PT-deficient mutants, SspE remains in a nuclease-inactive condition. No detectable DNA damage or growth defect was observed in PT-lacking, SspE-expressing cells. Upon PT detection, the GTPase activity of SspE is increased, thereby initiating a conformational alteration of SspE from a closed to an open state. This transition facilitates the dissociation of SspE from PT-modified self-DNA and activates the DNA nicking nuclease activity of the C-terminal DUF1524 domain of SspE, enabling SspE to damage phage DNA while simultaneously avoiding autoimmunity (27). In contrast, DndI remains persistently nuclease-active in PT-deficient cells, thereby resulting in DNA damage and impaired growth of bacterial cells. This suggests that although both SspE and DndI utilize hydrophobic cavities to sense PT markers, their reactivity to PT induces diverse enzymatic modulations, thereby exhibiting different self-versus-non-self DNA discrimination mechanisms.

In R-M systems, methylases almost fully modify the consensus sequences, using methyl groups to stop endonucleases from binding to self-DNA through steric hindrance while endonucleases are attacking foreign DNA (5). In contrast, only 10-15% of the 5′-GAAC-3′/5′-GTTC-3′ sequences within the *S. enterica* Cerro 87 genome DNA are PT-modified, and the distribution of PTs shows significant molecule-to-molecule heterogeneity even in the presence of nuclease-active DndFGH and DndI (44). In this study, we have revealed that when DndI reaches the 5′-G_PS_AAC-3′/5′-G_PS_TTC-3′ site, its ATPase activity is boosted, causing the DndI_NTD_ and DndI_CTD_ to come close to each other, which then facilitates the dissociation of DndI from the PT sites. However, how do the regions without PT modification defend themselves from self-restriction by DndI? Considering the fact that the DndFGH complex moves along DNA and adjusts its enzymatic activities upon reaching PTs to achieve the self-versus-nonself discrimination, our data imply that a yet-to-be-determined scanning mechanism of DndI, such as hopping or sliding along the DNA, might be involved in allowing DndI to not target non-PT-containing regions in self-DNA. Meanwhile, there is still a possibility that particular sequence motifs or secondary structures are implicated in preventing DndI from targeting non-PT-protected areas in self-DNA. Notably, a recent study has domonstrated that phages can evade the DndFGH defense by means of a Ser/Thr/Tyr protein kinase, which mediates multisite phosphorylation, thereby disrupting the defense function of DndFGH and enabling phage evasion (45). Given the intense phage-host arms race, it is possible that phages have potentially developed mechanisms to escape the DndI defense.

In conclusion, the study has found that DndI can work together with IscS-DndBCDE as an anti-phage defense system. Interestingly, the IscS-DndBCDE module is simultaneously used by DndFGH to fight against phages. This, along with the observation that *dndFGH* and *dndI* form an operon, emphasizes the complexity of the PT-based defense system throughout evolution.

## Supplementary Material

gkae1133_Supplemental_Files

## Data Availability

The coordinates and structure factors of DndI has been deposited in the Protein Data Bank under the accession numbers 9JFL.
