# Supplementary material for: Molecular basis of the phosphorothioation-sensing antiphage defense system IscS–DndBCDE–DndI
Source: Nucleic Acids Res. 2024 Nov 29;52(22):13594–604. doi: 10.1093/nar/gkae1133 (PMC11662687; doi:10.1093/nar/gkae1133)
Supplement: gkae1133_Supplemental_Files [file gkae1133_supplemental_files.zip › Tang et al_Supplementary Data 20241018.pdf]

Supplementary Information

Molecular and structural basis of the phosphorothioation-sensing antiphage defense system IscS-DndBCDE-DndI

Yaqian Tang[1,†], Dan Wu[1,2,†], Yueying Zhang[1], Xuan Liu[1], Hui Chu[1], Qian Tan[1], Lixu Jiang[3], Shi Chen[1,3,*], Geng Wu[4,*], Lianrong Wang[1,5,*]

Supplementary table S1. Strains, plasmids and phages used in this study

Name	Characteristics	Source or reference
**Strains**		
Salmonella enterica serovar Cerro 87	d(G_{PS}A/G_{PS}T)	PMID: 15019105
ΔdndB-H	Cerro 87 derivative, *dndB-H* in-frame deletion mutant	PMID: 20627870
ΔdndFGH	Cerro 87 derivative, *dndFGH* in-frame deletion mutant	Lab stock
iscS-dndBC$_{C280S}$DE-ΔdndFGH	Cerro 87 derivative, *dndFGH* in-frame deletion *dndC$_{C280S}$* mutant	This work
ΔdndBCDE-ΔdndFGH	Cerro 87 derivative, *dndBCDE* and *dndFGH* in-frame deletion	This work
E. coli BL21(DE3)	*F^- ompT gal dcm lon hsdS$_B$*(*rB^-mB^-*) λ(DE3 [*lacI lacUV5*-T7p07 *ind*1 *sam*7 *nin*5]) [*mal*B^+]K-12($λ^S$)	Novagen
E. coli JM109	*endA1 recA1 gyrA96 thi-1 hsdR17 (rk^-, mk+), relA1, supE44D (lac-proAB), [F'traD36, proAB, laqIqZΔM15]*	TransGen Biotech
**Plasmids**		
pBluescript II SK(+)	Cloning vector, Ampr	(1)
pPT1001	SK(+) derivative, carrying *dndBCDE* from Cerro 87	This work
pPT1002	SK(+) derivative, carrying *dndBCDEI* from Cerro 87	Lab stock
pPT1003	SK(+) derivative, carrying *dndBCDEI$_{Y25A}$* from Cerro 87	This work
pPT1004	SK(+) derivative, carrying *dndBCDEI$_{R87A}$* from Cerro 87	This work
pPT1005	SK(+) derivative, carrying *dndBCDEI$_{N541A}$* from Cerro 87	This work
pET28a	expressing vector, His-tag，Km^r	Novagen
pPT1006	pET28a derivative, expressing wild-type DndI from Cerro 87	This work
pPT1007	pET28a derivative, expressing DndI$_{Y25A}$ from Cerro 87	This work
pPT1008	pET28a derivative, expressing DndI$_{R87A}$ from Cerro 87	This work
pPT1009	pET28a derivative, expressing DndI$_{N541A}$ from Cerro 87	This work
pPT1010	pET28a derivative, expressing YFP-DndI-CFP	This work
pPT1011	pET28a derivative, expressing DndI$_{CTD}$	This work
pPT564	pET28a derivative, expressing both YFP and CFP	Lab stock
**Phages**		
PT1	Myoviridae, lytic, dsDNA	Lab stock
T4	*Myoviridae*, lytic, dsDNA	(2)
T1	*Siphoviridae*, lytic, dsDNA	(2)
EEP	*Siphoviridae*, lytic, dsDNA	(3)
λ	Siphoviridae, lytic or lysogenic, dsDNA	New England Biolabs
T7	Podoviridae, lytic, dsDNA	Lab stock

Supplementary table S2. Crystal parameters and refinement statistics of SeMet-DndI from *S. enterica* serovar Cerro 87

Structure	SeMet-DndI
**Data collection statistics**	
Beamline	BL17U1
Space group	$C222_1$
Wavelength (Å)	0.97916
a, *b*, *c* (Å)	103.74, 136.20, 225.99
α, β, γ (°)	90, 90, 90
Resolution (Å)	50.0-2.91 (3.01-2.91)
No. of molecule/asymmetric unit	2
No. of unique reflections	35,518
No. of all reflections	509,408
Completeness (%)	99.9 (100.0)
Redundancy	14.3 (14.3)
I/(I)	32.5 (1.50)
$R_{merge}{}^{a}$ (%)	49.7 (>100.0)
**Refinement statistics**	
Resolution (Å)	47.02-3.00 (3.08-3.00)
$R_{work}{}^{b}$ (%)	21.6 (29.3)
$R_{free}{}^{b}$ (%)	26.5 (28.6)
Completeness (%)	97.9 (84.1)
Overall B-factor (Å^2)	28.78
RMSD bond lengths (Å)	0.009
RMSD bond angle (°)	1.165
**Ramachandran plot**	
Res. in most favored regions	1072
Res. in additionally allowed regions	86
Res. in generously allowed regions	17

[a] $R_{merge} = \Sigma_{hkl}\Sigma_i|I(hkl)_i - \langle I(hkl)\rangle| / \Sigma_{hkl}\Sigma_i I(hkl)_i$, where $\langle I(hkl)\rangle$ is the mean intensity of the observations $I(hkl)_i$ of reflection hkl.

[b] $R_{\mathrm{work}} = \Sigma||F_{obs}|-|F_{calc}||/\Sigma|F_{obs}|$, where F_{obs} and F_{calc} are the observed and calculated structure-factor amplitudes, respectively. R_{free} was calculated as R_{work} using a randomly selected subset of ~5% of unique reflections not used for structure refinement.

Supplementary table S3. Primers used in this study

Primers	Sequences
DndI-F	tgccgcgcggcagccatatgATGAACGTTAAACCTGAGTA
DndI-R	tggtggtggtggtgctcgagCTAAGAGAAAGAAAAAACTT
DndI$_{Y25A}$-F	ACGCCTACAGCTCAAAGGGATTATTCATGGGAA
DndI$_{Y25A}$-R	ATCCCTTTGAGCTGTAGGCGTATAAAAAATATT
DndI$_{R87A}$-F	GGGCAACAAGCACTATCAACTATAGTA
DndI$_{R87A}$-R	AGTTGATAGTGCTTGTTGCCCATCAAC
DndI$_{N541A}$-F	CCAACCAGGGCTAATAAGAATGACAATAAACCT
DndI$_{N541A}$-R	ATTCTTATTAGCCCTGGTTGGATCTAAGAGAAC
DndI$_{CTD}$-F	ATGAACGTTAAAAATTTAACTGAAGGGGAATTGCTT
DndI$_{CTD}$-R	GTTAAATTTTTAACGTTCATaacttcatgttctctaaagt
**EMSA**	
70-bp DNA with 5′-GAAC-3′/5′-GTTC-3′	5′-GCTGCAACAGGATGCCAATAAGACCAAAAAAACCAGCCAGCAA GCAGAGAACGACGAGAGCTGGCAATCT-3′<br>3′-CGACGTTGTCCTACGGTTATTCTGGTTTTTTTGGTCGGTCGTTC GTCTCTTGCTGCTCTCGACCGTTAGA-5′
**FRET**	
CFP-F	CGggatccGTGAGCAAGGGCGAGGAGCT
CFP-R	AAGGAAAAAA gcggccgcCTTGTACAGCTCGTCCATGC
YFP-F	GGAATTCcatatgGTGAGCAAGGGCGAGGAGCT
YFP-R	TACTCAGGTTTAACGTTCATCTTGTACAGCTCGTCCATGC
DndI-YFP-F	GCATGGACGAGCTGTACAAGATGAACGTTAAACCTGAGTA
DndI-YFP-R	CGggatccAGAGAAAGAAAAAACTTTTA

Supplementary table S5. Efficiency of plating (EOP)

	PT1
ΔdndFGH	0.80 ± 0.04

EOP is calculated as the observed phage titer on *ΔdndB-I* divided by the phage titer on *ΔdndFGH*. All assays were performed at least three times.

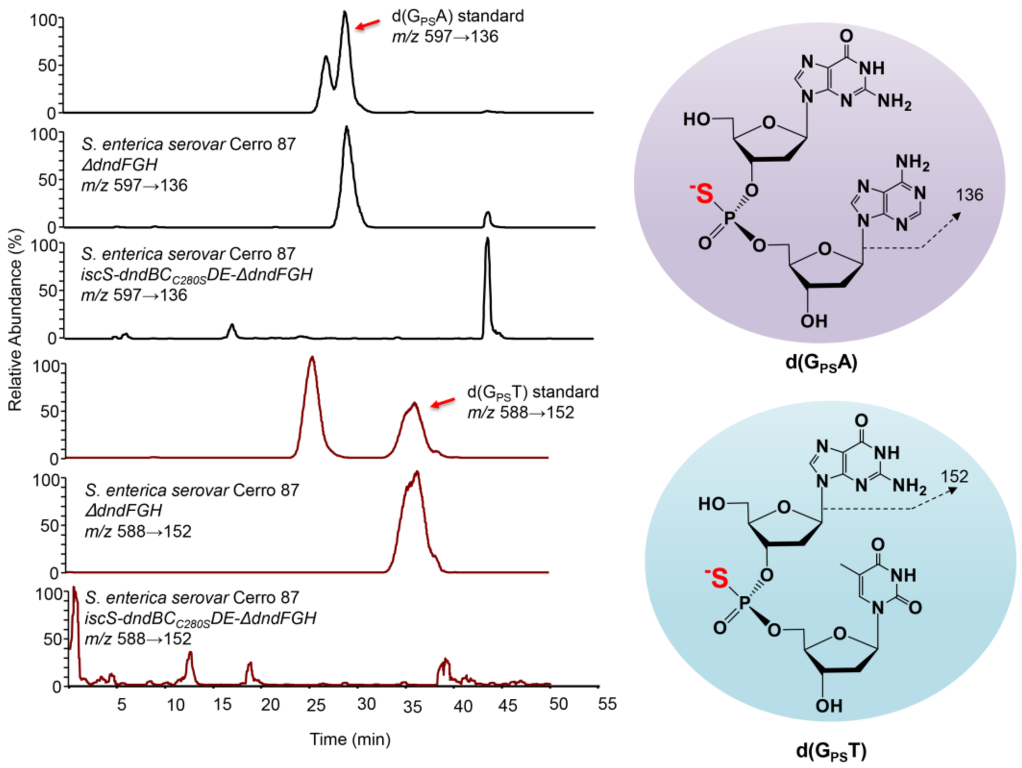

Supplementary Figure S1. LC-MS/MS-based detection of PT-modified d(G$_{PS}$A)/d(G$_{PS}$T) dinucleotides. The d(G$_{PS}$A)/d(G$_{PS}$T) modification was detected in *S. enterica* (*ΔdndFGH*). No d(G$_{PS}$A)/d(G$_{PS}$T) modification was detected in the *S. enterica* mutant (*iscS-dndBC$_{C280S}$DE-ΔdndFGH*). The fragmentation pattern of the d(G$_{PS}$A)/d(G$_{PS}$T) standard is shown in the inset.

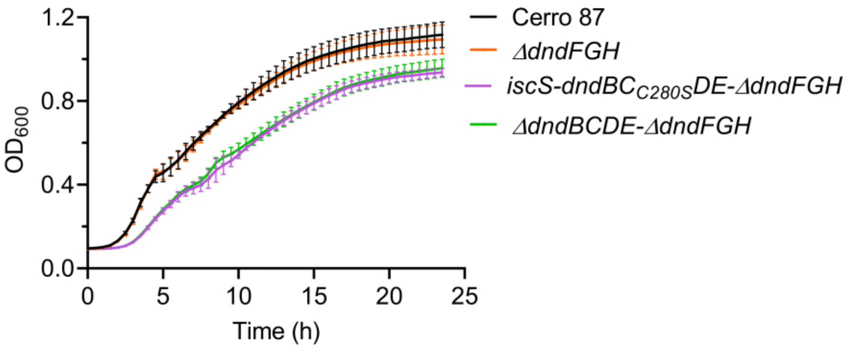

Supplementary Figure S2. The growth curves of *S. enterica* and mutants.

The DndI-expressing, PT-lacking mutants including *iscS-dndBC*$_{C280S}$*DE-ΔdndFGH* and *ΔdndBCDE-ΔdndFGH* displayed growth retardation.

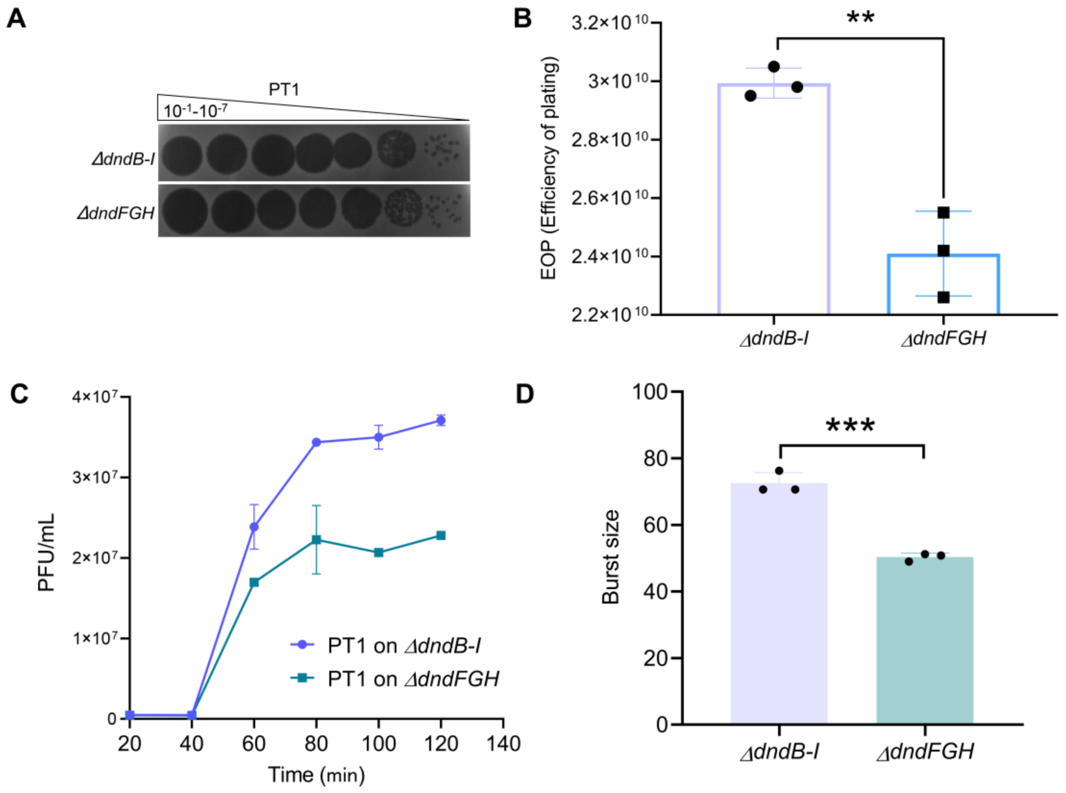

Supplementary Figure S3. Determination of the antiphage activity of IscS-DndBCDE-DndI in *S. enteria*. (A) Plaque assays of phage PT1 on IscS-DndBCDE-DndI-lacking and IscS-DndBCDE-DndI-expressing *ΔdndB-I and ΔdndFGH* cells, respectively. **(B)** The EOP values of phage PT1 infecting *ΔdndB-I and ΔdndFGH.* **(C and D)** One-step growth curves and average burst sizes of phage PT1 grown on *ΔdndB-I and ΔdndFGH*. The data represent the means ± SDs of three biological replicates. $^{**}P < 0.01$, $^{***}P < 0.001$.

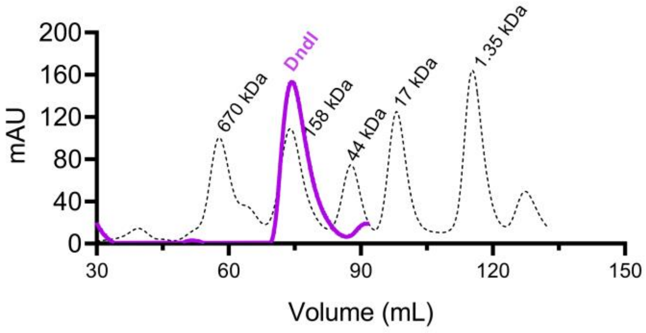

Supplementary Figure S4. The size exclusion chromatography analysis of DndI. The black curve and purple curve represent the UV absorption peak of standard proteins and DndI, respectively. Standard markers: thyroglobulin (670 kDa); gamma globulin (158 kDa); ovalbumin (44 kDa); myoglobin (17 kDa); vitamin B12 (1.35 kDa).

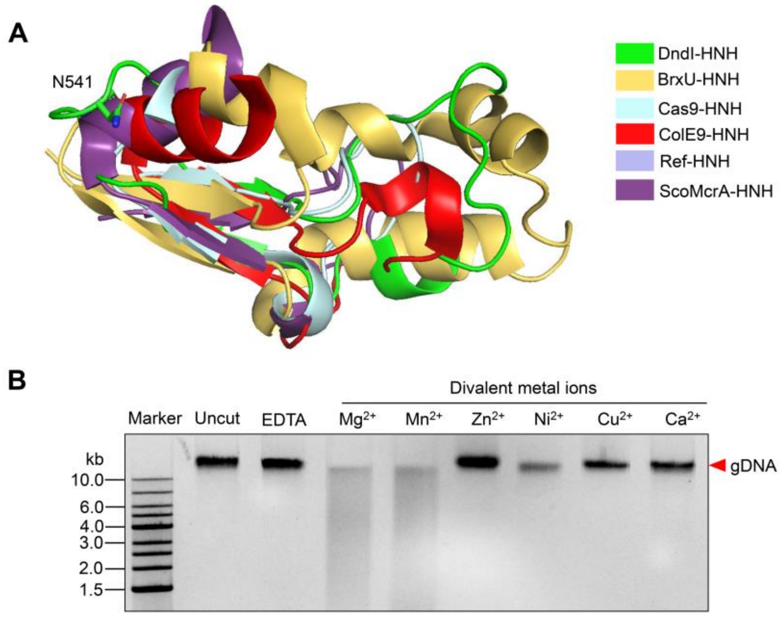

Supplementary Figure S5. Nuclease activity of DndI. (**A**) Structural comparison of the HNH motif in DndI with its homologous. Superimposed structures of the HNH motifs from DndI (PDB: 9JFL, green), BrxU (PDB: 7P9K, paleyellow), Cas9 (PDB: 6O56, palecyan), ColE9 (PDB: 1V14, red), Ref (PDB: 3PLW, lightblue) and ScoMcrA (PDB: 5ZMM, violetpurple). (**B**) Divalent cation requirements for the nicking activity of DndI towards genomic DNA of the PT-lacking *ΔdndB-I* mutant.

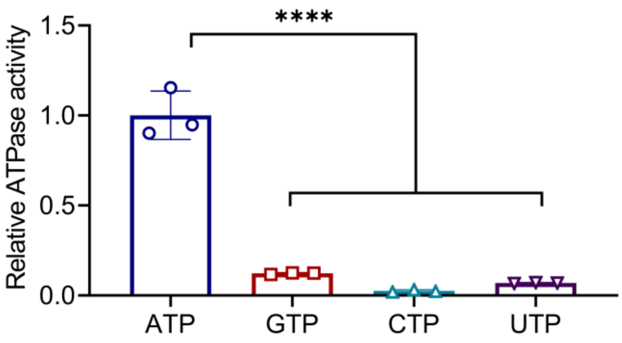

Supplementary Figure S6. Assessment of the NTPase activity of DndI. The hydrolysis activity of DndI towards different NTP substrates was evaluated. ****P < 0.0001.

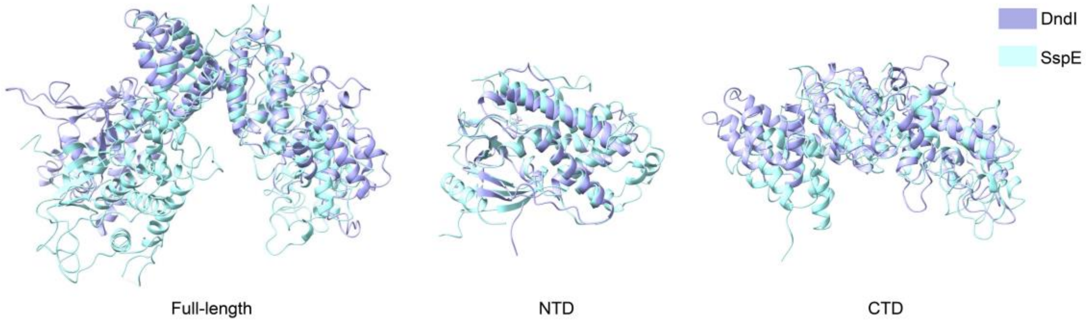

Supplementary Figure S7. Structural comparison of DndI and SspE. Showing the comparison of the full-length, NTD, and CTD of DndI (in lightpurple, PDB code: 9JFL) with those of SspE (in lightcyan, PDB code: 7DRS).

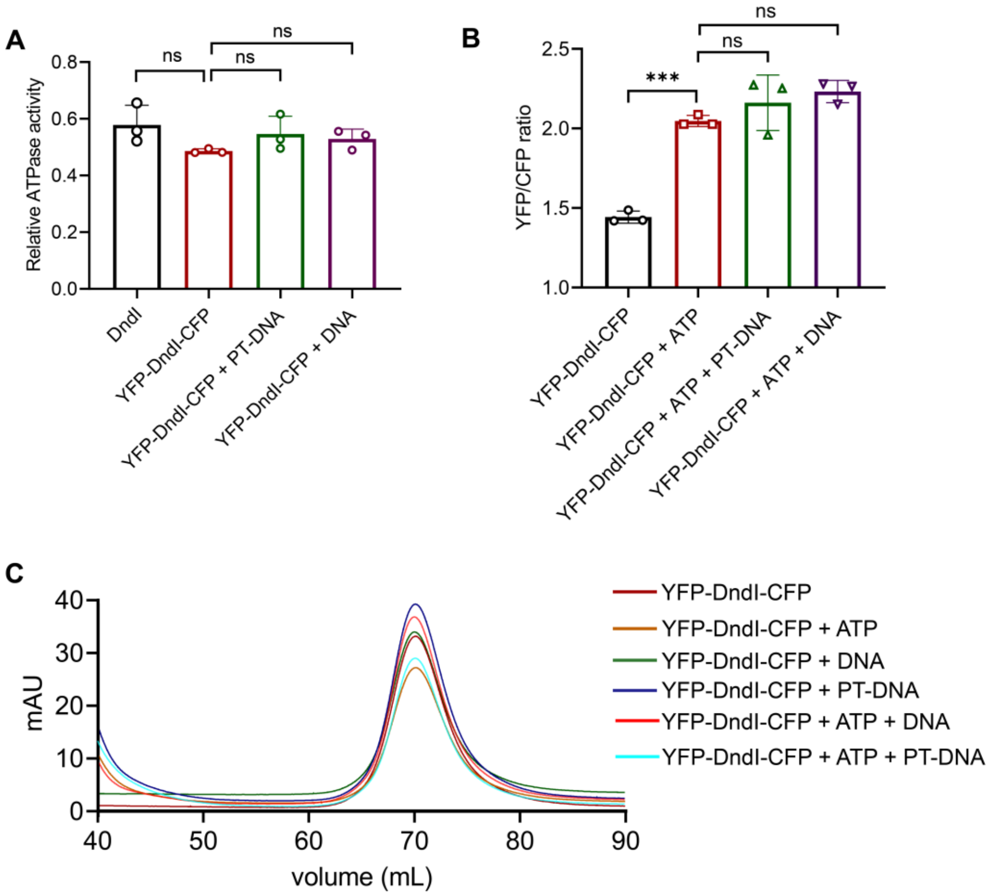

Supplementary Figure S8. Assessment of the ATPase activity and oligomeric state of YFP-DndI-CFP. (**A**) ATPase activities of YFP-DndI-CFP in the presence of 5′-G$_{PS}$AAC/G$_{PS}$TTC-3′-containing or PT-lacking pUC19 DNA. Statistical significance was calculated using unpaired two-sided Student's t tests. ****P* < 0.001. ns, not significant. (**B**) FRET analysis of YFP-DndI-CFP *in vitro*. FRET is represented as the emission ratio of YFP to CFP signals. (**C**) The oligomeric states of YFP-DndI-CFP were determined by gel filtration.

References

1. Xiong L, Liu S, Chen S, Xiao Y, Zhu B, Gao Y, Zhang Y, Chen B, Luo J, Deng Z, Chen X, Wang L, Chen S. (2019) A new type of DNA phosphorothioation-based antiviral system in archaea. *Nat Commun*, **10**, 1688.
2. Demerec M, Fano U. (1945) Bacteriophage-Resistant Mutants in *Escherichia Coli*. *Genetics*, **30**, 119-36.
3. Li S, Liu L, Zhu J, Zou L, Li M, Cong Y, Rao X, Hu X, Zhou Y, Chen Z, Hu F. (2010) Characterization and genome sequencing of a novel coliphage isolated from engineered *Escherichia coli*. *Intervirology*, **53**, 211-20.
